# Gestational and lactational exposure to gossypol alters the testis transcriptome

**DOI:** 10.1186/s12864-020-6487-2

**Published:** 2020-01-17

**Authors:** Helder Louvandini, Patricia S. Corrêa, Rocío Amorín, Lihe Liu, Egon H. Ieda, Carolina R. Jimenez, Siu M. Tsai, Concepta M. McManus, Francisco Peñagaricano

**Affiliations:** 1 Department of Animal Sciences, University of Florida, Gainesville, FL 32611 USA; 2 Laboratory of Animal Nutrition, Centre for Nuclear Energy in Agriculture, University of São Paulo, Piracicaba, SP 13400-970 Brazil; 3 Laboratory of Molecular and Cell Biology, Centre for Nuclear Energy in Agriculture, University of São Paulo, Piracicaba, SP 13400-970 Brazil; 4 Institute of Biology, University of Brasília, Brasília, Federal District 70910-900 Brazil; 5 University of Florida Genetics Institute, University of Florida, Gainesville, FL 32611 USA

**Keywords:** Cottonseed, Fetal programming, Male reproduction, Maternal nutrition

## Abstract

**Background:**

Reproductive capacity can be altered by challenges experienced during critical periods of development, including fetal development and early neonatal life. Gossypol is a polyphenolic compound, commonly found in cotton seeds, that impairs male reproduction. Here, we investigated whether the exposure to gossypol in utero and during lactation alters male reproductive function in sheep. From conception until 60 days postpartum, ewes were randomly assigned to a control diet or a gossypol-rich diet based on cottonseed. Lamb testicles were removed at 60 days of age and subjected to RNA-sequencing.

**Results:**

Lambs derived from the maternal cottonseed diet showed significantly lower growth and lower testis weight as a proportion of the total body weight, and reduced testosterone levels. In addition, the testis transcriptome was significantly altered by the maternal cottonseed diet. Most of the altered genes are directly implicated in testis development and sperm biology, cell communication, iron ion metabolism, calcium homeostasis and signaling, among other functions. Interestingly, network analysis revealed that exposure to gossypol significantly disturbed coexpression patterns among spermatogenesis-related genes, suggesting a disruption in coregulation mechanisms.

**Conclusions:**

Our findings provide evidence that maternal exposure to gossypol alters male reproductive function in the offspring, with potential lasting or lifelong negative consequences.

## Background

The use of cotton by-products in animal nutrition is limited by the presence of gossypol, a toxic molecule. Gossypol, a phenolic compound (C_30_H_30_O_8_), is found in the roots and foliage of the cotton plant, but its greatest concentration is in the seeds. In general, this secondary plant compound has negative effects on mammalian cell metabolism [1]. Gossypol toxicity is related to its ability to bind macromolecules before and after absorption. Two gossypol forms have been identified, free (toxic) and bound. The bound form is synthesized via covalent bonds between free gossypol and the free epsilon-amino groups from lysine and arginine [2]. This reaction reduces the availability of these amino acids, particularly lysine. In addition, free gossypol also binds minerals, specially iron which inhibits the absorption of this metal, affecting the erythropoiesis [1].

Gossypol affects both female and male gametogenesis and also embryo development. Female exposure to gossypol has been associated with irregular and longer estrous cycles, lower levels of estradiol, reduced number of ovarian follicles, and decreased pregnancy rate [3–5]. The negative effect of gossypol on male reproduction has been reported in several studies, including degeneration of spermatocytes in hamsters [6], decreased sperm count and motility, increased abnormal sperms, and reduced testosterone concentration in rats [7–9], and reduced sperm production, reduced sperm motility, and increased sperm abnormalities in bulls [10, 11]. Interestingly, the effect of gossypol on male fertility is both dose- and time-dependent: in effective doses, gossypol causes infertility by affecting sperm motility and damaging the germinal epithelium; however the adverse effects are reversible when gossypol in no longer ingested [11, 12].

There is growing evidence that intrauterine stimulus or insults can affect offspring sexual development. Maternal nutrition is considered a major intrauterine environmental factor in fetal development. Indeed, it is now recognized that maternal nutrition plays a key role in programming offspring reproductive capacity [13]. For instance, in utero protein restriction reduces Sertoli cells, alters sperm motility and counts, and increases abnormal sperm morphology in adult male rats [14]. In addition, maternal protein restriction during lactation reduces testicular weight and decreases testicular aromatase expression in male rat offspring, indicating potential harm for future germ cell development and reproductive function [15]. Little is known, however, about the potential effects of gestational and lactational exposure to gossypol on the offspring sexual development. As such, the main objective of this study was to investigate the effect of maternal cottonseed supplementation from conception to weaning on the development of male reproductive function in sheep. Specifically, we hypothesized that a maternal diet rich in gossypol during gestation and throughout lactation would alter both testis development and testis gene expression in the offspring.

## Results

### Body measurements

Maternal diets did not affect lamb birth weights (Table 1). After birth, however, lambs exposed to gossypol showed lower milk intake, lower body weight gain and lower gonadosomatic index when compared to lambs exposed to a control diet (*P*-value ≤0.05, Table 1). These findings suggest that gestational and lactation exposure to gossypol impacts the development of the offspring, which in turn may have long-term consequences.

**Table 1:** Body weight (kg), milk intake (mL/day) and gonadosomatic index (GSI %) from lambs exposed to either a control or a gossypol-rich (cottonseed) maternal diet

Variables	Maternal Diets		SEM	*P*-value
	Control	Cottonseed		
Birth Weight	4.39	3.62	1.14	0.99
Final Weight	15.7	11.0	0.81	0.004
Milk Intake	989	764	215	0.042
GSI	0.049	0.032	0.006	0.019

### Hemogram analysis

The hemogram analysis revealed that lambs exposed to gossypol in utero and through lactation showed significantly lower red blood cell count, lower hematocrit, and also lower hemoglobin compared to lambs derived from the maternal control diet (*P*-value ≤0.05, Table 2).

**Table 2:** Hemogram analysis from lambs exposed to either a control or a gossypol-rich (cottonseed) maternal diet

Variables	Maternal Diets		SEM	*P*-value	Reference^a^
	Control	Cottonseed			
Red blood cell (× 10^6^/μL)	9.85	9.47	0.19	0.049	9–15
Hematocrit (%)	39.28	37.27	0.85	0.020	27–45
Hemoglobin (g/dL)	11.49	10.97	0.25	0.039	9–15
White blood cell (×10^3^/mL)	8.17	8.06	0.47	0.81	4–8

^a^Reference values in sheep according to Byers and Kramer [16]

### Hormone analysis

**Fig. 1:**
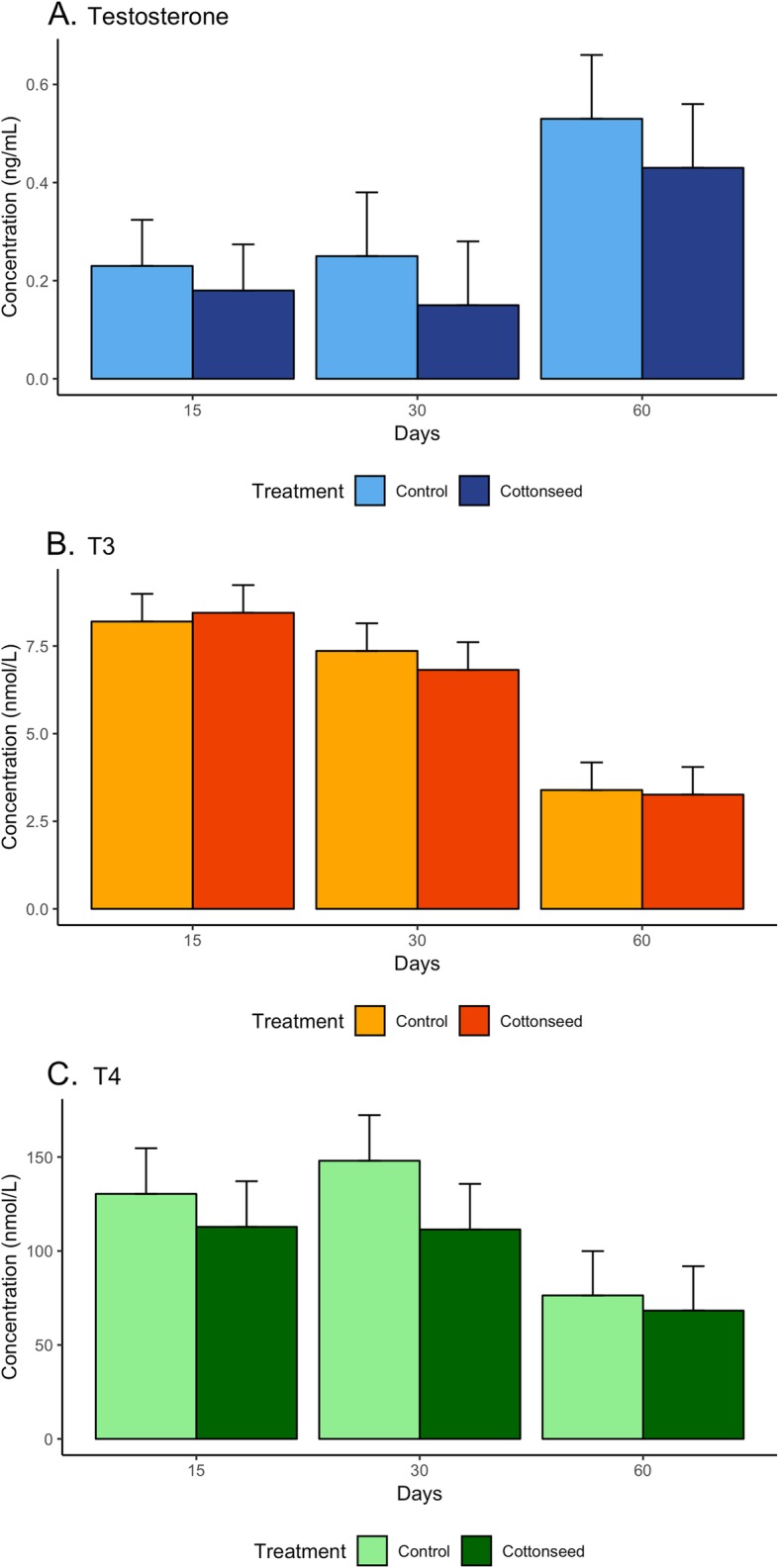
Concentration of hormones (**a**) Testosterone, (**b**) Triiodothyronine [T_3_], and (**c**) thyroxine [T_4_] in days 15, 30 and 60 after birth. Lighter colors indicate lambs exposed to a maternal control diet while darker colors refer to lambs exposed to a maternal cottonseed diet. Maternal diets did not affect hormone concentrations while there were significant time effects (*P*-value ≤ 0.05), i.e. testosterone levels increased at day = 60 while both T_3_ and T_4_ levels decreased at day = 60

The levels of testosterone increased while the levels of both T3 and T4 decreased across time, from birth to weaning (*P*-value ≤0.05, Fig. 1). Interestingly, the levels of testosterone were systematically lower in lambs derived from the gossypol-rich maternal diet. Although these differences were not statistically significant, they hold biological importance as testosterone plays key roles in male sexual development.

### RNA-sequencing

**Fig. 2:**
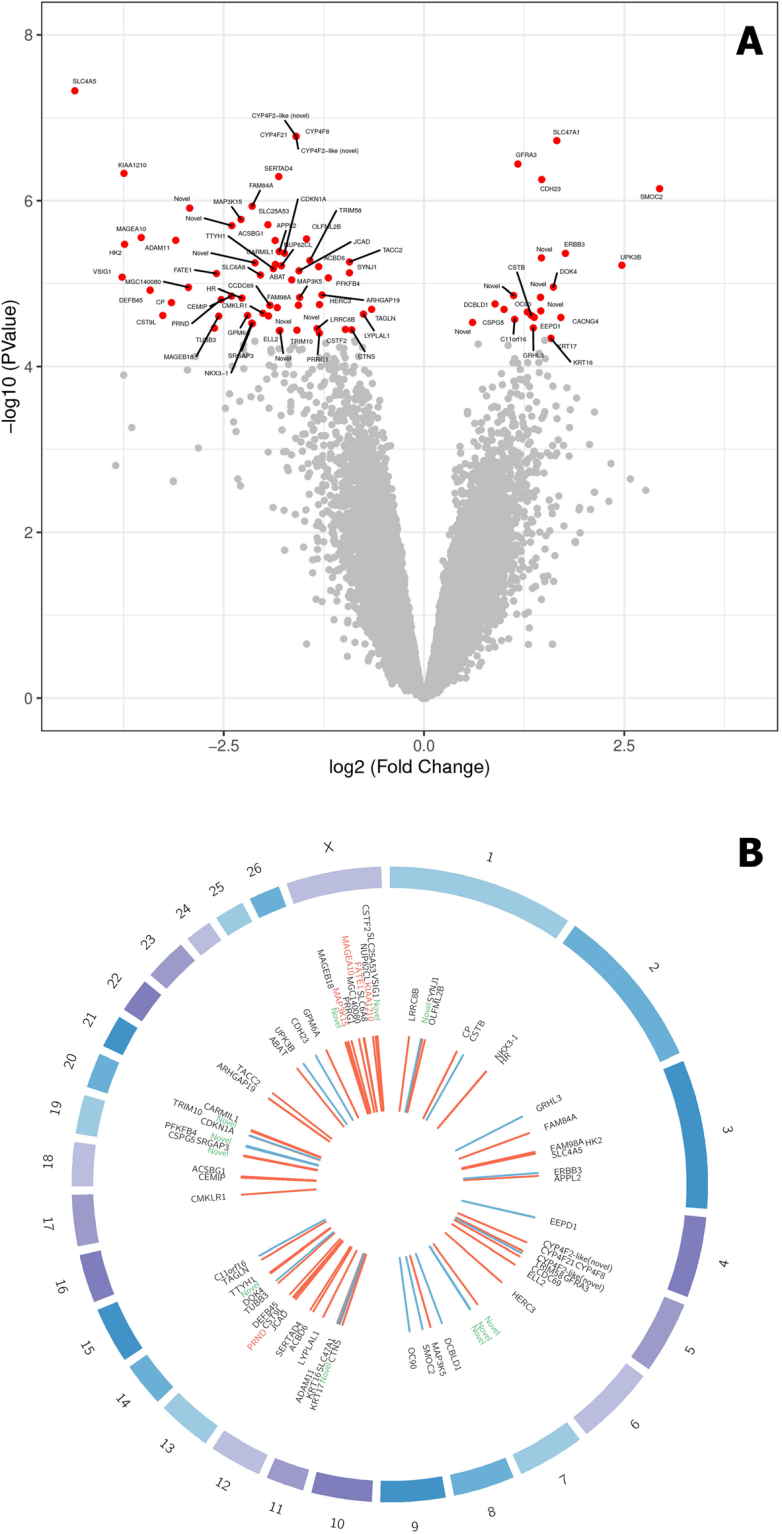
Changes in testis transcriptome between maternal diets. **a** Volcano plot showing gene expression changes in lambs exposed to either a control diet (CT, *n* = 9) or a cottonseed diet (GO, n = 9). The x-axis shows the magnitude of the change in gene expression while the y-axis shows the statistical significance of the change in gene expression. **b** Circos plot showing differentially expressed genes. The outer ring shows the chromosomes in the *Ovis aries* genome; the middle ring shows the name of all differentially expressed genes (FDR ≤ 0.01): in green are novel genes and in red are genes selected for qRT-PCR validation; the inner ring shows tiles depicting differentially expressed genes, red are downregulated genes while blue are upregulated genes due to gossypol exposure

The RNA-sequencing of the testis transcriptome yielded approximately 24 million sequencing reads per sample. Roughly 81% of the reads were mapped to the Oar_v3.1 sheep genome assembly using the software Tophat (Additional file 1). A total of 18,326 genes were tested for differential expression. Controlling false discovery rate (FDR) at 1%, a total of 84 genes showed differential expression between maternal diets (*P*-value ≤4.6e-05, Fig. 2). Additional file 2 shows the full list of significant genes, including Ensembl gene ID, log2-fold-change, log2-counts-per-million, *P*-value and q-value. Most of the significant genes (62 out of 84) were down-regulated in lambs derived from the maternal gossypol-rich diet. Interestingly, many of these down-regulated genes are directly implicated in testis development, spermatogenesis, and sperm physiology, such as cystatin-9-like (*CST9L*), NK3 homeobox 1 (*NKX3.1*), a disintegrin and metallopeptidase domain 11 (*ADAM11*), V-set and immunoglobulin domain containing 1 (*VSIG1*), MAGE family member B18 (*MAGEB18*), leucine rich repeat containing 8 VRAC subunit B (*LRRC8B*), acrosomal protein KIAA1210 (*KIAA1210*), mitogen-activated protein kinase 15 (*MAP3 K15*), solute carrier family 4 member 5 (*SLC4A5*), fetal and adult testis expressed 1 (*FATE1*), and prion like protein doppel (*PRND*), among others. On the other hand, 22 out of 84 differentially expressed genes were up-regulated due to gossypol exposure. Many of these significant genes are directly implicated in xenobiotic metabolism, such as solute carrier family 47 member 1 (*SLC47A1*) or intracellular calcium homeostasis and signaling, such as SPARC related modular calcium binding 2 (*SMOC2*), erb-b2 receptor tyrosine kinase 3 (*ERBB3*), calcium voltage-gated channel auxiliary subunit gamma 4 (*CACNG4*), cadherin related 23 (*CDH23*), and otoconin 90 (*OC90*).

### Gene expression validation

The expression of five genes directly implicated in male reproduction, namely *KIAA1210*, *MAP3 K15*, *SLC4A5*, *FATE1* and *PRND*, was validated using qRT-PCR. The RNA-Seq analysis revealed that these genes were down-regulated in the maternal cottonseed diet. In the same way, results from qRT-PCR clearly indicated that the expression of these five genes was significantly decreased due to gossypol exposure (*P*-value ≤0.05, Additional file 3).

### Gene-set enrichment analysis

**Fig. 3:**
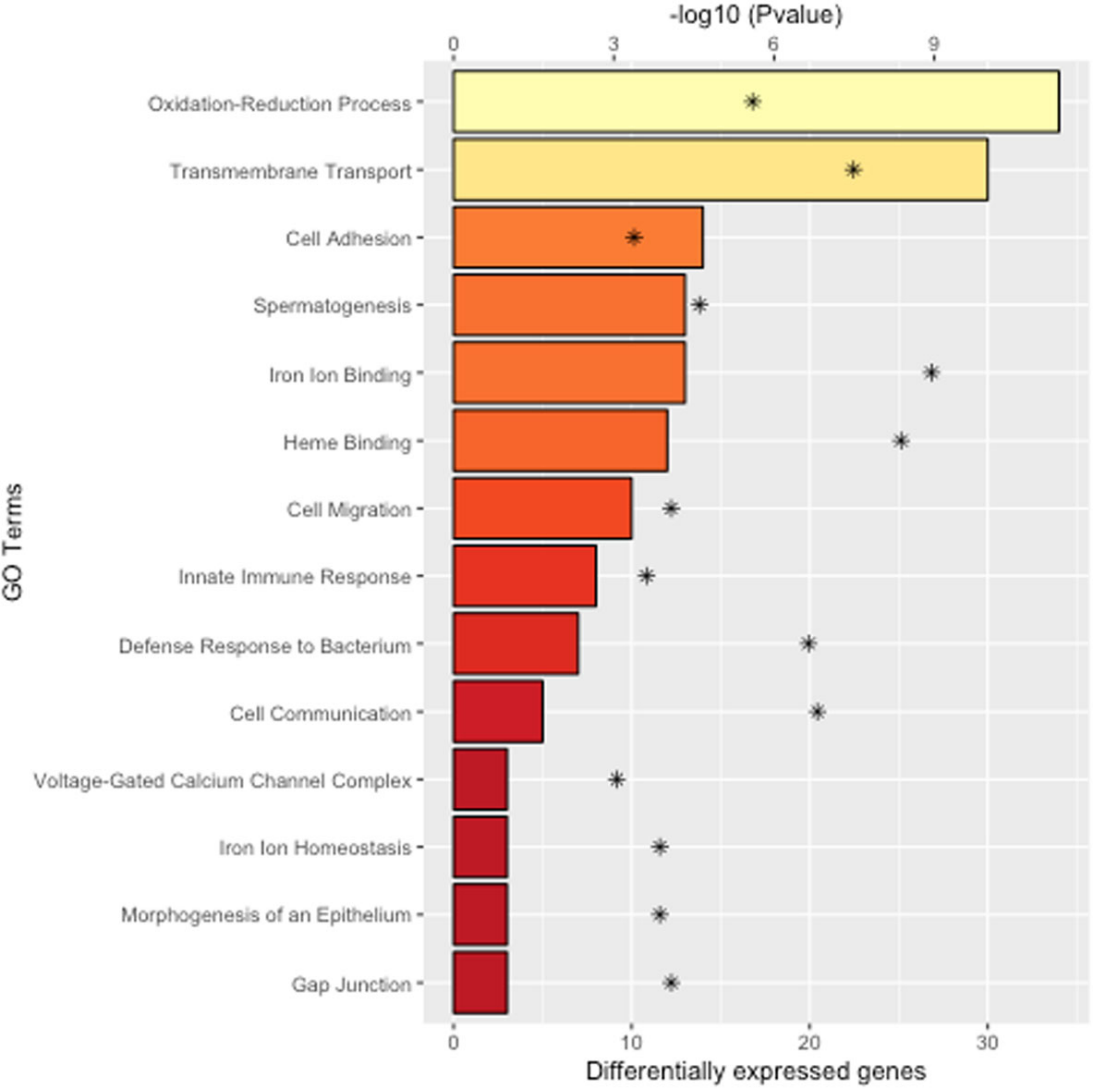
Gene Ontology terms significantly enriched with differentially expressed genes between maternal control and maternal cottonseed diets. The bottom x-axis shows the number of differentially expressed genes in each GO term while the top x-axis shows the significance of the enrichment as -log_10_ (*P*-value)

An enrichment analysis, also known as overrepresentation analysis, was performed in order to gain additional insight into the biological processes that could be impacted by gossypol in the lamb testicles. Figure 3 displays a set of Gene Ontology terms that were significantly enriched with differentially expressed genes. Some of these functional terms are closely related to testis function, such as *spermatogenesis* (GO:0007283). In addition, some terms are directly implicated in ion iron metabolism, such as *ion iron binding* (GO:0005506) and *heme binding* (GO:0020037), and also calcium homeostasis, such as and *voltage-gated calcium channel complex* (GO:0005891). Interestingly, many significant terms are associated with epithelial tissue integrity and homeostasis, such as *cell communication* (GO:0007154), *gap junction* (GO:0005921), and *morphogenesis of an epithelium* (GO:0002009), suggesting that gossypol exposure might impair the highly specialized epithelial tissue found in the testicles. Finally, some terms were closely related to the immune function, such as *defense response to bacterium* (GO:0042742) and *innate immune response* (GO:0045087). Additional file 4 shows the full list of significant GO terms, including GO ID, GO name, number of genes, number of differentially expressed genes, and Fisher’s *P*-value.

### Gene coexpression network analysis

**Fig. 4:**
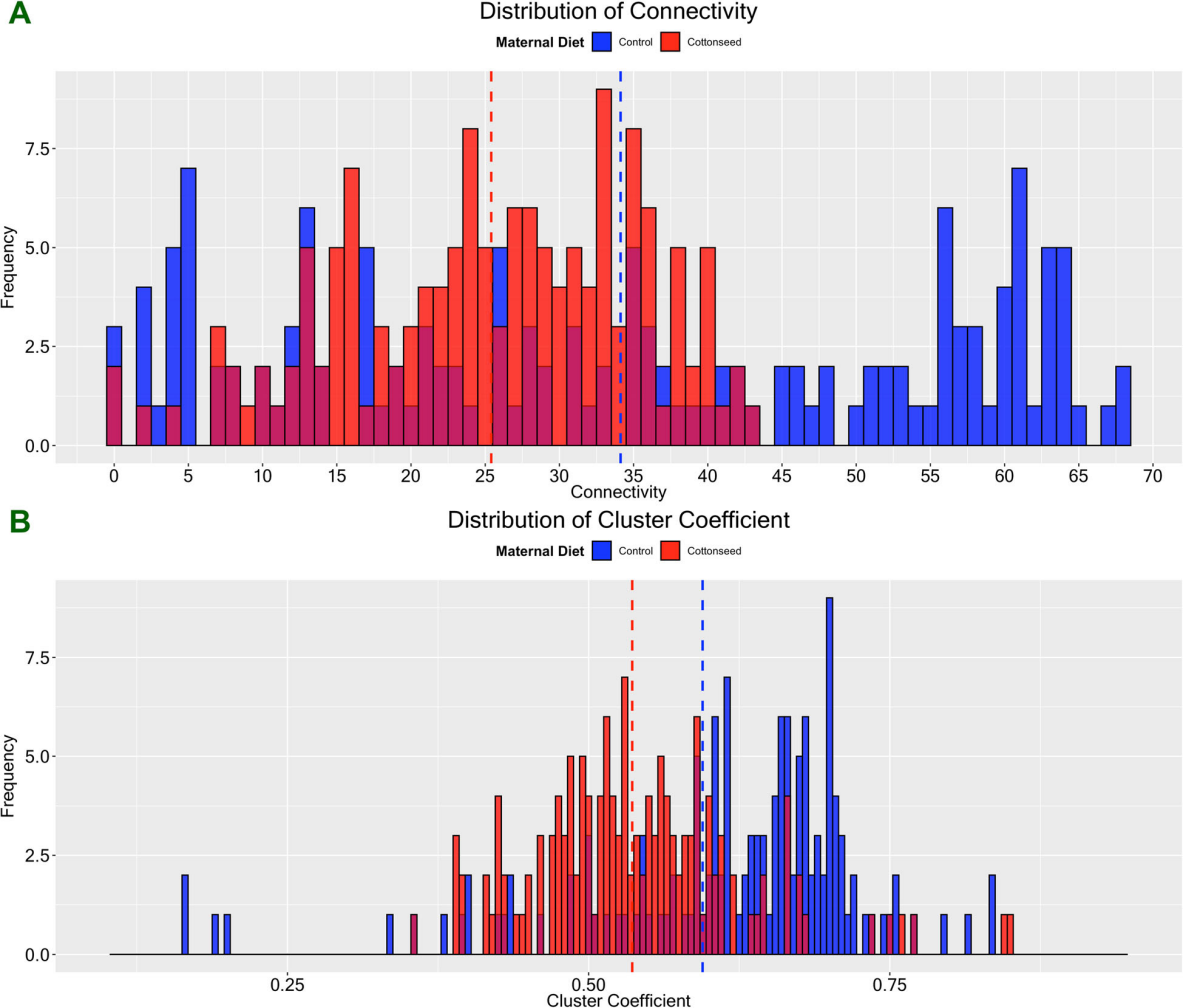
Spermatogenesis coexpression network analysis: distributions of node connectivity and node clustering coefficient between maternal diets. **a** Node connectivity; (**b**) Node clustering coefficient. Control diet is represented in blue while gossypol-rich cottonseed diet is represented in red. Treatment means are marked by the dashed vertical lines: the blue line is for the control diet and the red line is for the gossypol-rich cottonseed diet

The potential impact of gossypol on spermatogenesis was further investigated using gene coexpression network analysis. The goal was to investigate the preservation of the coexpression network between maternal diets using the expression of 145 spermatogenesis-related genes. Figure 4 shows the distribution of the node connectivity and node cluster coefficient, two classical network preservation statistics, in each maternal diet. Interestingly, the exposure to gossypol in utero and throughout lactation caused noticeable changes in the coexpression patterns of genes directly implicated in spermatogenesis. Indeed, the maternal gossypol-rich diet altered the spermatogenesis network decreasing both gene connectivity and gene clustering, suggesting a clear disruption in gene coexpression patterns.

## Discussion

Nutritional programming describes the effects that nutritional changes during key moments of development have on future animal performance. The present study was specially designed to evaluate the effects of maternal cottonseed supplementation, a feed rich in gossypol, on male reproductive function. Our results showed that exposure to gossypol in utero and throughout lactation led to significant changes in testis development and gene expression. To the best of our knowledge, this is the first study that reports programming of sexual development and male reproductive function due to maternal cottonseed nutrition.

The exposure to gossypol in utero and throughout lactation negatively impacts growth and testis development. Lambs derived from the maternal cottonseed diet had not only lower body weight at weaning but also significantly lower gonadosomatic index, indicating smaller testicles in relation to the total body mass. This is a clear indication that the gestational and lactational exposure to gossypol impairs normal development of the testicles, which in turn could have lasting or lifelong consequences on male reproductive function. Several studies in male rats have reported that exposure to gossypol caused degeneration of seminiferous tubules and reduced testosterone concentration [7–9]. Interestingly, we found that, although not statistically significant, testosterone levels were consistently lower in lambs from dams supplemented with cottonseed. Given that testosterone is produced by the Leydig cells, interstitial cells found adjacent to the seminiferous tubules, lower testosterone levels can be explained either by the reduced testicle mass and/or a direct impact of gossypol on the physiology of the Leydig cells [17, 18].

Maternal exposure to gossypol directly impacted the testis transcriptome of the offspring. Most significantly altered genes were found down-regulated in the maternal gossypol-rich diet. Notably, many of these down-regulated genes, such as *CST9L*, *FATE1*, *NKX3.1*, *ADAM11*, *VSIG1*, *KIAA1210*, *CYP4F21*, *SLC4A5*, and *PRND*, are closely related to male fertility and sperm biology. For instance, gene *CST9L* is a testis-specific protein, member of the superfamily of cysteine peptidase inhibitors, that has a relevant role in tissue reorganization during early testis development [19]. Gene *FATE1* is a X-linked gene predominantly expressed in fetal and adult testis implicated in both testicular development and germ cell differentiation [20]. Gene *NKX3.1* is a homeobox transcription factor which exhibits prostate and testis specific expression, and is essential for normal testis function given that its loss of expression is highly associated with testicular germ cell tumorigenesis [21]. Gene *ADAM11* encodes a member of the disintegrin and metalloprotease protein family, membrane-anchored proteins that have been implicated in a variety of biological processes involving cell-cell and cell-matrix interactions, including gametogenesis and fertilization [22, 23]. Gene *VSIG1* is a member of the junctional adhesion molecule family, is primarily expressed in the stomach and testis, and plays important roles during spermatogenesis [24]. Gene *KIAA1210* is predominantly expressed in testis and plays an active role in the acrosome reaction, a crucial process during sperm-oocyte fusion [25]. Gene *CYP4F21* encodes a cytochrome P450 enzyme which is involved in the biosynthesis of 20-hydroxy-PGE_1_ and 20-hydroxy-PGE_2_, two specific and presumably physiologically important compounds excreted by the male vesicular glands [26]. Gene *SLC4A5* encodes a member of the sodium bicarbonate cotransporter family, is highly expressed in the testis and plays a key role in regulating sodium and bicarbonate transport, influencing testis intracellular, extracellular, and interstitial pH [27]. Gene *PRND* encodes a membrane glycoprotein that is found predominantly in testis and has a crucial function during the late steps of spermatogenesis [28]. Overall, these results indicate that prenatal and lactational exposure to gossypol alters the expression of many genes involved in testicular function.

Although several studies have shown the toxicity of gossypol in male reproduction, the precise mechanisms of gossypol action in the testicles have not yet been fully elucidated. Of special interest, *spermatogenesis* (GO:0007283) was identified as one of the most impacted functional gene-sets by the maternal cottonseed diet (Fig. 3, Fisher’s *P*-value ≤0.01). Indeed, a total of 13 genes directly implicated in the spermatogenesis process showed differential expression between maternal diets. Notably, the gene coexpression network analysis revealed that the maternal exposure to gossypol significantly altered the coexpression patterns of spermatogenesis-related genes, suggesting that gossypol might disrupt gene coregulation mechanisms. Recently, Lim et al. proposed that gossypol induces negative effects on mice testis function by reducing cell viability, mitochondrial membrane potential, and the expression of many genes related to development and maturation of sperm cells [29]. Spermatogenesis is a multi-step process that involves multiple cellular events including cell-cell interactions, cell migration, apoptosis and differentiation. Interestingly, we found that functional gene-set terms such as *cell communication* (GO:0007154), *cell adhesion* (GO:0007155), *gap junction* (GO:0005921), *cell migration* (GO:0016477), and *negative regulation of apoptotic signaling pathway* (GO:2001234) were significantly impacted by gossypol exposure.

It is well-documented that free gossypol is a highly reactive compound that readily binds to iron, forming a gossypol-iron complex, which limits iron bioavailability and inhibits iron absorption. Here, lambs derived from the maternal gossypol-rich diet showed significantly lower red blood cells, hematocrit, and hemoglobin, compared to lambs from maternal control diet. Note that gossypol-mediated iron deficiency impairs heme synthesis, which in turn limits hemoglobin synthesis. Interestingly, the negative effect of gossypol on iron homeostasis was also revealed by the gene expression data. Indeed, some of the most significant gene-sets in the pathway analysis were closely related to iron metabolism, such as *iron ion binding* (GO:0005506), *heme binding* (GO:0020037), and *iron ion homeostasis* (GO:0055072). The vast majority of the genes in these functional terms were down-regulated in lambs from the maternal cottonseed treatment, providing further evidence that maternal exposure to gossypol negatively altered iron ion transport, signaling, and metabolism in the offspring.

There is growing evidence that gossypol also alters intracellular calcium homeostasis and signaling. Indeed, several studies have shown that gossypol causes a rapid increase in cytoplasmic calcium as a consequence of release of calcium from endoplasmic reticulum stores, and subsequent calcium influx through calcium release-activated channels [30–32]. Interestingly, we found that many genes up-regulated in lambs derived from the maternal gossypol-rich diet, such as *CDH23* and *CACNG4*, are directly implicated in calcium homeostasis. For instance, gene *CDH23* encodes a member of the cadherin superfamily, which comprises calcium-dependent cell-cell adhesion glycoproteins involved in diverse biological processes including calcium ion transport and regulation of cytosolic calcium ion concentration [33]. Similarly, gene *CACNG4* encodes a transmembrane protein that plays an active role in the regulation of calcium ion transmembrane transport [34]. Furthermore, the gene-set enrichment analysis identified functional terms, such as *transmembrane transport* (GO:0055085), *positive regulation of release of sequestered calcium ion into cytosol* (GO:0051281), and *voltage-gated calcium channel complex* (GO:0005891), that are directly implicated in calcium ion homeostasis. Overall, our findings provide additional evidence that gossypol alters calcium metabolism, which in turn can mediate some of its detrimental effects.

## Conclusions

Our findings provide evidence that maternal cottonseed supplementation during the gestational and lactational period alters male reproductive function in the offspring. To the best of our knowledge, this is the first study showing that gossypol exposure during fetal development and early neonatal life can severely impact sexual development. The observed changes in testis development and testis gene expression suggest that exposure to gossypol in utero and during lactation can have lasting or lifelong consequences on male fertility.

## Methods

### Ethics statement

All the animal procedures used in this study were approved by the Committee on Animal Research and Ethics (008/2015) of the Center of Nuclear Energy in Agriculture, University of São Paulo, Brazil. All experiments were performed in accordance with relevant guidelines and regulations.

### Animals, experimental design, and maternal diets

Santa Inês ewes (*Ovis aries*) from the Center for Nuclear Energy in Agriculture at the University of São Paulo, Brazil, were used in a completely randomized design in order to evaluate the effect of gestational and lactational exposure to cottonseed-derived gossypol on testis development and testis gene expression in lambs. From conception until 60 days postpartum, ewes were individually supplemented with a control diet based on corn and soybean or a gossypol-rich diet based on cottonseed [35]. Ewes had ad libitum access to hay and forage (pastures of *Panicum maximum cv. Aruana*). The diets were elaborated to meet the requirements of protein and metabolizable energy for gestation and lactation.

### Lamb measurements and blood collection

A total of 18 male lambs, 9 from control and 9 from gossypol-rich maternal diets, were used in this study. Lambs were kept in collective pens with ad libitum access to water, tifton hay, mineral salt, and concentrate (70% corn and 30% soybean meal) provided in creep feeding. Milk intake was estimated according to the method proposed by Robinson et al. [36]. Blood samples were collected on days 0, 3, 7, 15, 30, 45 and 60 after birth by puncture of the jugular vein, using vacuum tubes either with or without anticoagulant (EDTA). A hematological analyzer (Davol® poch-100 iV, São Paulo, Brazil) was used to determine red blood cell count (RBC), white blood cell count (WBC), hemoglobin concentration (Hg), hematocrit (Ht), and platelet count (Plc). Blood samples without EDTA were centrifuged for 10 min at 1310 g and 4 °C for serum separation and stored at − 20 °C for further analysis of testosterone (T), thyroxine (T_4_) and triiodothyronine (T_3_) on days 15, 30 and 60. Hormone levels were analyzed by radioimmunoassay (RIA) following the instructions of iodine ^125^I RIA kits Beckman coulter® (T/IM1087, T_3_/IM1699 and T_4_/IM1447), Praha, Czech Republic. The entire right and left testicles were removed at 60 days of age. Lambs were sedated with a 0.1 mg/kg dose intramuscular xylazine hydrochloride, followed by local anesthesia containing 2 mL of 2% lidocaine hydrochloride with epinephrine. The gonadosomatic index (GSI) for each lamb was calculated as the weight of both testicles divided by total body weight. The left testicle was frozen in nitrogen liquid and stored in − 80 °C for subsequent RNA-sequencing.

### Statistical analysis

The statistical analysis of the body weight, milk intake, GSI, blood parameters, and hormone data was performed using SAS® 9.2 software (SAS Institute Inc., Cary NC, USA/2014). For the response variables measured only once, namely milk intake and GSI, the effect of the maternal diets was assessed using PROC Anova. On the other hand, body weight records, hemogram parameters, and hormone levels were evaluated several times, and hence the effect of maternal nutritional treatments was evaluated using the procedure of repeated measures in PROC Mixed.

### RNA extraction, library preparation and sequencing

Total RNA was extracted from 18 testicles derived from 18 lambs, 9 from maternal control diet and 9 from maternal gossypol-rich diet. RNA extraction was performed using Trizol® reagent, and quality of the obtained RNA was determined using a nanodrop spectrophotometer (OD260/OD280) to determine purity, as well as an agarose gel electrophoresis to observe potential RNA degradation and contamination. The RNA integrity was assessed using an Agilent Bioanalyzer, with RNA integrity number (RIN) values between 8 and 10. All the RNA-sequencing procedures were performed by Novogene Bioinformatics Technology Co., Ltd. (Beijing, China). Sequencing libraries were prepared using a Poly-A tail capture method and sequenced with Illumina’s HiSeq 3000. Whole-genome transcriptome sequencing data can be accessed by GEO with the accession number GSE133811.

### RNA-seq data analysis

RNA-sequencing reads were tested for quality before and after trimming using the software FastQC (version 0.11.7, Babraham Bioinformatics, UK). Trimming was conducted with the software Trim Galore (version 0.4.4, Babraham Bioinformatics, UK) using the following parameters: *--quality* 20, *−-clip_R1* 10, *−-three_prime_clip_R1* 20, and *--length* 50. Note that reads that became shorter than 50 bp were discarded. A first round of single-end alignment was performed on the Oar_v3.1 sheep genome assembly using the software Tophat2 (version 2.1.1) with the option *--coverage-search* to enable search of novel junctions [37]. Resulting junction files were then merged together and used for a second round of mapping as a way to fully maximize the identification of novel transcripts. A maximum of 2 mismatches were allowed and reads that mapped to more than 40 genomic locations were discarded. The resulting alignments were provided to software Cufflinks (version 2.2.1) in order to construct transcript models. Sample assemblies were merged together with the Oar_v3.1 sheep genome assembly annotation file using Cuffmerge (Cufflinks, version 2.2.1) in order to combine novel transcripts with known annotated transcripts; this strategy maximizes the overall quality of the final assembly. Read counting was performed with htseq (version 0.6.1p1) using the final transcriptome assembly with the option *intersection-nonempty* [38]. Differentially expressed genes between maternal diets were detected using the *R* package edgeR (version 3.20.9) with default parameters [39].

### Validation of differentially expressed genes

Five differentially expressed genes were chosen for validation of RNA-Seq results: *KIAA1210*, *MAP3 K15*, *MAGEA10*, *FATE1* and *PRND*. The same RNA samples used for RNA-Seq were used here for gene expression validation using quantitative real-time PCR (qRT-PCR). Total RNA was quantified through spectrometry using Nanodrop 2000c (Thermo Fisher Scientific) and 1000 ng of total RNA were treated with 1 U of DNase I (Thermo Scientific) in a 10 μL reaction containing 1 μL + DNase-RNase-Free water at 37 °C for 10 min. The cDNA was prepared using the Maxima First Strand cDNA Synthesis kit (Thermo Fisher, Waltham, MA). Reactions were run using StepOnePlus system (Applied Biosystems, Foster City, CA). Gene *GAPDH* was chosen as internal control because of its stable expression across all RNA-Seq samples. The association between normalized gene expression values (ΔCt) and maternal diets was tested using a likelihood ratio test [40]. The relative gene expression values were calculated using the 2^-ΔΔCt^ method [41].

### Gene-set enrichment analysis

The significant enrichment of Gene Ontology (GO) terms with differently expressed genes between maternal nutritional treatments was analyzed using Fisher’s exact test, a test of proportions based on the cumulative hypergeometric distribution. Differentially expressed genes that showed FDR ≤ 0.05 and had ENSEMBL annotations were tested against the background set of all expressed genes with ENSEMBL annotations. The assignment of genes to GO terms was performed using the function *getBM* from the *R* package biomaRt (v 2.36.1). The Fisher’s exact test was implemented using the function *fisher.test* in the *R* software.

### Gene coexpression network analysis

Network modeling analysis was performed to further characterize the process of spermatogenesis that could be directly impacted by maternal gossypol exposure. A total of 145 genes that showed expression in the RNA-Seq analysis and belong to GO *spermatogenesis* (GO:0007283) were considered in this analysis. For each maternal nutritional treatment, a *correlation matrix R* = (*r*_*ij*_) with dimensions 145 × 145 was constructed using Pearson correlation coefficients. Each correlation matrix was then translated into an *adjacency matrix A* = (*a*_*ij*_), an 145 × 145 matrix with entries either 0 or 1. Here, if |*r*_*ij*_| ≥ 0.5 and *P*-value ≤0.05, then *a*_*ij*_ = 1, otherwise *a*_*ij*_= 0. Finally, for each maternal diet, an unweighted network was constructed based on *adjacency matrix* where two genes *i* and *j* were either connected (*a*_*ij*_ = 1) or disconnected (*a*_*ij*_ = 0). The structure and topology of each network was evaluated using node *connectivity* and node *cluster coefficient* [42]. The *connectivity k*_*i*_ of gene *i*, defined as ${k}_i=\sum \limits_{j\ne i}{a}_{ij}$, measures the connection strength of gene *i* with all the other genes. The *cluster coefficient c*_*i*_ of gene *i*, defined as,

$$ {c}_i=\frac{\sum_{j\ne i}{\sum}_{k\ne i}{a}_{ij}{a}_{jk}{a}_{ki}}{{\left({\sum}_{j\ne i}{a}_{ij}\right)}^2-\sum \limits_{j\ne i}{\left({a}_{ij}\right)}^2} $$

represents the local density of a network with 0 ≤ *c*_*i*_ ≤ 1. All these analyses were performed using the *R* package Weighted Correlation Network Analysis (WGCNA, version 1.66) [43].

## Supplementary information

**Additional file 1.** Sequencing and mapping statistics.

**Additional file 2.** List of significant genes.

**Additional file 3.** qRT-PCR validation results.

**Additional file 4.** List of significant GO terms.

## Data Availability

Sequencing data can be accessed through NCBI GEO with accession number GSE133811.

## Abbreviations

FDR: False discovery rate
GO: Gene ontology
RNA-Seq: RNA sequencing
