# Supplementary figures and images for: Gestational and lactational exposure to gossypol alters the testis transcriptome
Source: BMC Genomics. 2020 Jan 17;21:59. doi: 10.1186/s12864-020-6487-2 (PMC6969474; doi:10.1186/s12864-020-6487-2)

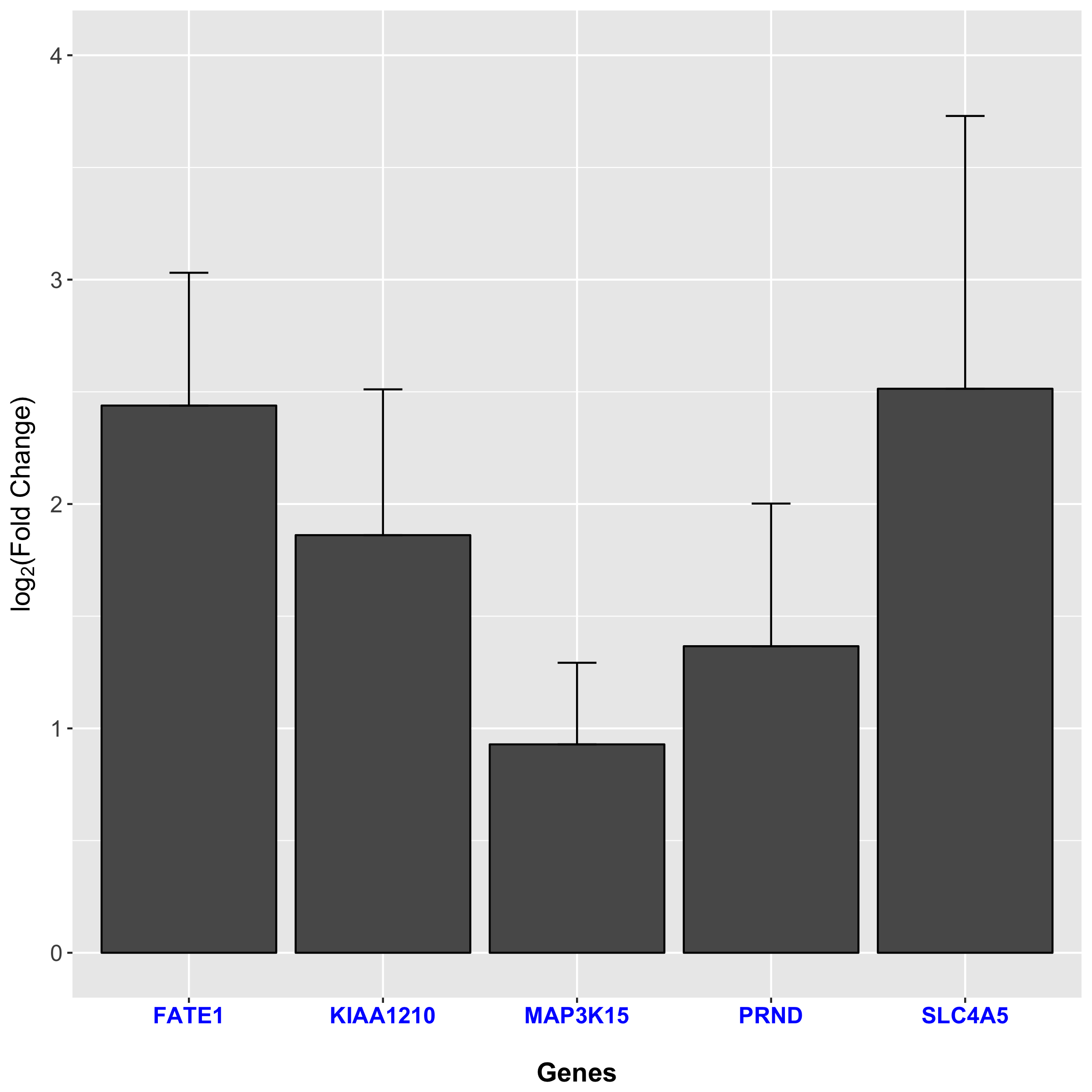

Supplement: Supplementary file 3 — Additional file 3. qRT-PCR validation results. [file 12864_2020_6487_MOESM3_ESM.tiff]
